# Daily oral administration of low‐dose methotrexate has greater antirheumatic effects in collagen‐induced arthritis rats

**DOI:** 10.1111/jphp.12752

**Published:** 2017-05-31

**Authors:** Aoi Koyama, Aki Tanaka, Hideto To

**Affiliations:** ^1^ Department of Medical Pharmaceutics Graduate School of Medicine and Pharmaceutical Sciences for Research University of Toyama Toyama Japan

**Keywords:** arthritis, dosing schedule, low dose, methotrexate, myelosuppression

## Abstract

**Objectives:**

Methotrexate (MTX) is administered once or thrice weekly to patients with rheumatoid arthritis (RA). Even though RA continually progresses, MTX is not administered daily. Therefore, we investigated whether the daily administration of a low dose of MTX inhibits the progression of arthritis in collagen‐induced arthritis (CIA) rats.

**Methods:**

Methotrexate was orally administered once weekly, thrice weekly and once daily to CIA rats, and arthritis scores were measured.

**Key findings:**

When the same dose of MTX was administered, the exacerbation of arthritis was inhibited significantly more in the once‐daily group than in the other groups. When the dose in the once‐daily group was reduced to one‐fourth that of the current standard dosing method, arthritis scores were markedly lower in the once‐daily group than in the once and thrice‐weekly groups.

**Conclusions:**

The daily administration of a low dose of MTX not only maintained normal levels that estimated adverse effects but also suppressed the progression of arthritis significantly more than the current standard dosing method. The results indicate that the reconsideration of dosing schedules based on the characteristics of MTX will lead to more effective RA therapy than that currently used in clinical practice.

## Introduction

Rheumatoid arthritis (RA) is a chronic autoimmune disorder that affects various tissues and also causes inflammation in synovial joints.^1^, ^2^ Although RA is more common in 30‐ to 50‐year‐old females than males, ethnicity, the location of residence and climate are not important factors affecting the prevalence of RA.^3^ Due to the progression of joint destruction with RA, joint function, activity of daily life and quality of life are reduced in patients with RA. Decreases in joint function may be restored with therapy, but become irreversible with the progression of joint destruction. Prominent joint destruction occurs within 2 years of the onset of RA.^4^ Therefore, appropriate treatments from the early stage of RA contribute to preventing the progression of its pathology.

The development of biological disease‐modifying antirheumatic drugs (Bio‐DMARDs) has led to marked improvements in treatment of RA in recent years.^5^, ^6^ Although patients with RA may achieve complete remission with Bio‐DMARDs, difficulties are associated with the continuation of this treatment for the following reasons: infections related to declines in immune function occur because the blood concentration half‐life of Bio‐DMARDs is extremely long, and as Bio‐DMARDs are very expensive, their use is restricted to a limited number of patients with RA because therapeutic costs are too high for many patients.

Methotrexate (MTX) is the most commonly administered antirheumatic drug worldwide and exerts dose‐dependent therapeutic effects.^7^ Recommended starting dosage schedules of MTX are in a 7.5 mg single dose (once weekly) or 2.5 mg divided dose (three doses every 12 h once a week). Accordingly, MTX requires a washout period to prevent adverse effects from developing in patients with RA. Nevertheless, the MTX dose administered to patients with RA is approximately one‐hundredth that in cancer chemotherapy. Even though inflammatory responses are common events in patients with RA, MTX requires a washout period. Therefore, the current standard dosing method may result in the aggravation of joint inflammation.

A large number of basic and clinical studies have been performed with the aim of improving the therapeutic effects of MTX. Increases in the dosage administered and appropriate dosing schedules have achieved greater efficacy than that of the current dosing method, though the effectiveness may have limitations to achieve complete remission.^7^, ^8^ This may be one of the reasons why MTX is not more regularly administered in a week. The daily administration of MTX is not recommended because it has been suggested to increase the risk of adverse effects. The development of adverse effects with the daily administration of MTX has been attributed to the total dose per week being in excess of that with the current standard dosing method. In cancer chemotherapy, adverse effects are exacerbated when the total dose per period of time is increased.^9^, ^10^, ^11^ However, the efficacy and toxicity of the daily administration of a low dose of MTX at an identical total dosage in 1 week to that of the current standard dosing method have not yet been evaluated. The aggravation of arthritis may be inhibited without the development of severe adverse effects when a low dose of MTX is administered once every day. Although this idea is a simple and fundamental approach, it has been almost not studied as a RA treatment using MTX in basic and clinic, so it seems to be a novel research. Therefore, we herein investigated whether the daily administration of a low dose of MTX improves arthritis while maintaining safety in collagen‐induced arthritis (CIA) rats.

## Materials and Methods

### Animals

Female Lewis rats (7 weeks old) were purchased from Japan SLC. Rats were housed three rats/cage under standardized light–dark cycle conditions (lights on and off at 7:00 and 19:00, respectively) with free access to food and water. The protocol of the experiments was reviewed and approved in advance, and experiments with animals were performed in accordance with the Guide for Animal Experimentation from the Committee for Animal Experiments at the University of Toyama, which is in accordance with the Guidelines for Proper Conduct of Animal Experiments from the Science Council of Japan.

### Induction of collagen‐induced arthritis

Bovine type II collagen (CII) (Cosmo Bio Co., Ltd., Tokyo, Japan) and Freund's incomplete adjuvant (Becton, Dickinson, Tokyo, Japan) were mixed, and an emulsion was prepared at a concentration of 1 mg/ml in CII. Rats were intradermally sensitized at ten sites (per 100 μl) on day 0 by the administration of 1 mg CII. Seven and 10 days later, rats received an intradermal booster injection of one‐tenth of the volume used for sensitization. Sensitization was performed under anaesthesia.

### Evaluation of arthritis scores

Arthritis scores were recorded every day after the first immunization. A previously described arthritis scoring system^12^ was used that evaluated individual joints and weighed the severity of arthritis by joint size as follows: (1) for the interphalangeal joints, each of the four lateral digits in the hind legs was scored as 0 or 1 (0 = no arthritis and 1 = arthritis present), and (2) for the ankle and midfoot joints, each was scored on a scale of 0–4 (0 = normal, 1 = minimal swelling, 2 = moderate swelling, 3 = severe swelling and 4 = severe swelling and non‐weight bearing). The macroscopic score was expressed as a cumulative value for all paws, with a maximum possible score of 32.

### Preparation of methotrexate

Methotrexate (Wako Pure Chemical Industries, Ltd., Osaka, Japan) was dissolved in sodium bicarbonate (Otsuka Pharmaceutical Co., Ltd., Tokyo, Japan). The final concentrations in each dosing group were 0.05 mg/ml (0.1 mg/kg), 0.125 mg/ml (0.25 mg/kg), 0.25 mg/ml (0.5 mg/kg), 0.5 mg/ml (1.0 mg/kg), 1.165 mg/ml (2.33 mg/kg) and 3.5 mg/ml (7.0 mg/kg). MTX was perorally (p.o.) administered to rats by gavage at 2 ml/kg.

### Experiment

Table 1 was shown study design in this study.

**Table 1:** Study designs

Experiment	Dosing schedule	Dosage (mg/kg per week)	Dosage (mg/kg per time)	Treated days after the first immunization (efficacy) or Treated days (Toxicity)	Number of mice	Observations
1–1	Control	0	0	8–14	12	Arthritis score (Figure 1)
	Once weekly	7	7	8	12	
	Thrice weekly	7	2.33	8 (twice daily), 9	12	
	Once daily (1.0 mg/kg)	7	1	8–14	12	
1–2	Control	0	0	1–21	6	Arthritis score (Figure 2)
	Once weekly	7	7	1, 8, 15	6	
	Thrice weekly	7	2.33	1 (twice daily), 2 8 (twice daily), 9 15 (twice daily), 16	6	
	Once daily (1.0 mg/kg)	7	1	1–21	6	
2	Control	0	0	1–21	18	Arthritis score (Figure 3)
	Once weekly	7	7	1, 8, 15	6	
	Thrice weekly	7	2.33	1 (twice daily), 2 8 (twice daily), 9 15 (twice daily), 16	18	
	Once daily (0.1 mg/kg)	0.7	0.1	1–21	6	
	Once daily (0.25 mg/kg)	1.75	0.25	1–21	6	
	Once daily (0.5 mg/kg)	3.5	0.5	1–21	6	
	Once daily (1.0 mg/kg)	7	7	1–21	6	
3	Normal	0	0	1–21	6	Type II collagen antibody (Figure 4)
	CIA	0	0	1–21	6	
	Thrice weekly	7	2.33	1 (twice daily), 2 8 (twice daily), 9 15 (twice daily), 16	6	
	Once daily (0.25 mg/kg)	1.75	0.25	1–21	6	
	Once daily (1.0 mg/kg)	7	7	1–21	6	
4	Control	0	0	0–20	6	WBC counts (Figure 5) ALT and BUN levels (Table 2)
	Thrice weekly	7	2.33	0–20	6	
	Once daily (0.25 mg/kg)	1.75	0.25	0 (twice daily), 1 7 (twice daily), 8 14 (twice daily), 15	6	
	Once daily (1.0 mg/kg)	7	7	0–20	6	

#### Experiment I: Influence of the dosing schedule of methotrexate on antirheumatic effects in collagen‐induced arthritis rats

Methotrexate was orally administered at a dose of 7.0 mg/kg once weekly (*n* = 6 or 12), 2.33 mg/kg thrice weekly (*n* = 6 or 12) and 1.0 mg/kg once daily (*n* = 6 or 12) for 3 weeks from the next day after the first or second immunization (day 1 or 8). The total dose per week was equal among all dosing groups. Sodium bicarbonate was administered to the control group (*n* = 6 or 12). Arthritis scores were measured every day after the first immunization.

#### Experiment II: Influence of dosing‐dependent antirheumatic effects after the daily administration of methotrexate to collagen‐induced arthritis rats

Methotrexate was orally administered at a dose of 0.1, 0.25, 0.5 and 1.0 mg/kg daily from the next day (day 1) after the first sensitization (*n* = 6, respectively). MTX was orally administered at a dose of 7.0 mg/kg once weekly (*n* = 6) and 2.33 mg/kg thrice weekly (*n* = 18). Sodium bicarbonate was administered to the control group (*n* = 18). Arthritis scores were measured every day after the first immunization.

#### Experiment III: Methotrexate dosing schedule‐dependent serum type II collagen antibody levels

Methotrexate was orally administered at a dose of 2.33 mg/kg thrice weekly and 0.25 or 1.0 mg/kg once daily for 3 weeks from the next day (day 1) after the first sensitization (*n* = 6, respectively). Sodium bicarbonate was administered to the normal group and CIA groups (*n* = 6, respectively). Blood samples were drawn from the tail vein of each rat on day 22 after the first immunization, and type II collagen antibody levels were measured using a serum type II collagen antibody level kit (Chondrex Inc., WA, USA).

#### Experiment IV: Influence of the dosing schedule of methotrexate on adverse effects in normal rats

Methotrexate was orally administered at a dose of 2.33 mg/kg thrice weekly, and 0.25 or 1.0 mg/kg once daily for 3 weeks to rats (*n* = 6, respectively). Sodium bicarbonate was administered to the control group (*n* = 6). Blood was collected from the caudal vein on days 3, 7, 17 and 21, and leucocyte numbers were measured using a leucocyte counter (Sysmex, Kobe, Japan).

To investigate alanine aminotransferase (ALT) and blood urea nitrogen (BUN) concentrations, blood samples were drawn from the tail vein of each rat on days 7, 14 and 21. Serum concentrations of ALT and BUN were measured using a transaminase CII test (Wako Pure Chemical Industries Ltd., Japan) and urea nitrogen B‐test (Wako Pure Chemical Industries Ltd., Osaka, Japan).

### Statistical analyses

Data were recorded as the mean ± standard deviation (SD) or median. Statistical analysis was performed by SPSS statistics ver.23. Arthritis score was treated as nonparametric data. Intragroup *post hoc* testing was performed using the Mann–Whitney *U*‐test with Bonferroni correction after Kruskal–Wallis test. To analyse type II collagen antibody, leucocyte count, ALT and BUN, groups were compared by a one‐way analysis of variance (ANOVA) and differences between groups were analysed using Scheffé's test. A probability level of <0.05 was considered to be significant.

## Results

### Influence of the dosing schedule of methotrexate on antirheumatic effects in collagen‐induced arthritis rats

**Figure 1:**
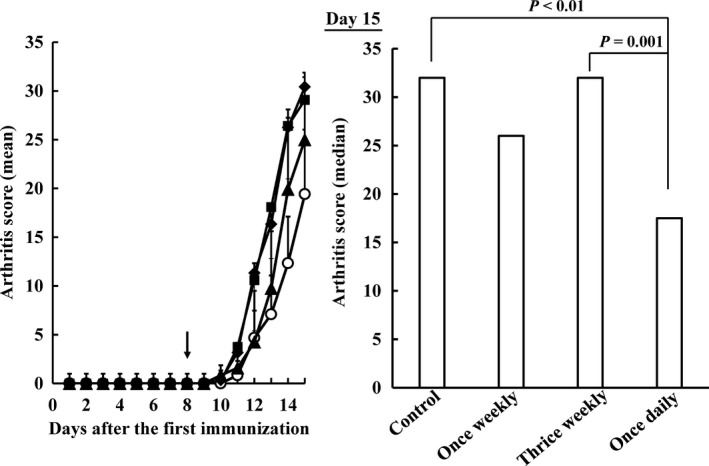
Influence of the dosing schedule of methotrexate on arthritis scores in collagen‐induced arthritis rats after the second immunization. methotrexate was orally administered at a dose of 7.0 mg/kg once weekly (closed triangles), 2.33 mg/kg thrice weekly (closed diamonds) and 1.0 mg/kg once daily (open circles) for 3 weeks. The total dose per week was equal among all dosing groups. Sodium bicarbonate was administered to the control group (closed square). Each value represents the mean ± SD (*n* = 12). An arrow showed start date of methotrexate administration. Arthritis scores were significantly lower in the once‐daily group than in the 2.33 mg/kg thrice‐weekly and control groups on day 15 (*P* < 0.01, respectively).

When CIA rats were administered MTX from the next day after the second sensitization (day 8), arthritis scores (mean ± SD) on day 15 were 29.1 ± 4.8 in the control group, 30.4 ± 3.3 in the thrice‐weekly group, 25.0 ± 6.9 in the once‐weekly group and 19.4 ± 6.6 in the once‐daily group (Figure 1). The aggravation of arthritis was inhibited significantly more in the once‐daily group than in the thrice‐weekly and control groups (*P* < 0.01 and *P* = 0.001, respectively).

**Figure 2:**
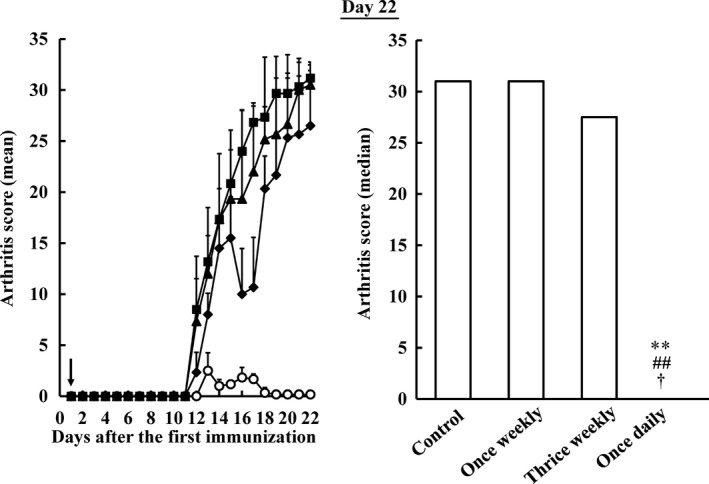
Influence of the dosing schedule of methotrexate on arthritis scores in collagen‐induced arthritis rats after the first immunization. methotrexate was orally administered at a dose of 7.0 mg/kg once weekly (closed triangle), 2.33 mg/kg thrice weekly (closed diamond) and 1.0 mg/kg once daily (open circle) for 3 weeks. Sodium bicarbonate was administered to the control group (closed square). Each value represents the mean ± SD (*n* = 6). An arrow showed start date of methotrexate administration. **P < 0.01 vs the control group, ^##^ *P* < 0.01 vs the once‐weekly group, ^†^ *P* < 0.05 vs the thrice‐weekly group. The inhibition of arthritis was the greatest in the once‐daily group among all the dosing groups tested.

Figure 2 shows arthritis scores when MTX was orally administered to each group for 3 weeks from the next day after the first immunization (day 1). Arthritis in the control and once‐weekly groups continuously deteriorated from day 11. The aggravation of arthritis was temporarily improved in the thrice‐weekly group over that in the control group. However, no significant differences were observed in arthritis scores between the control and thrice‐weekly groups on day 22. On the other hand, arthritis was hardly observed in the once‐daily group during the dosing period, and the exacerbation of arthritis was inhibited significantly more in the once‐daily group than in the other dosing groups on day 22 (*P* < 0.05 and *P* < 0.01, respectively).

### Influence of dose‐dependent antirheumatic effects of methotrexate administered once daily to collagen‐induced arthritis rats

**Figure 3:**
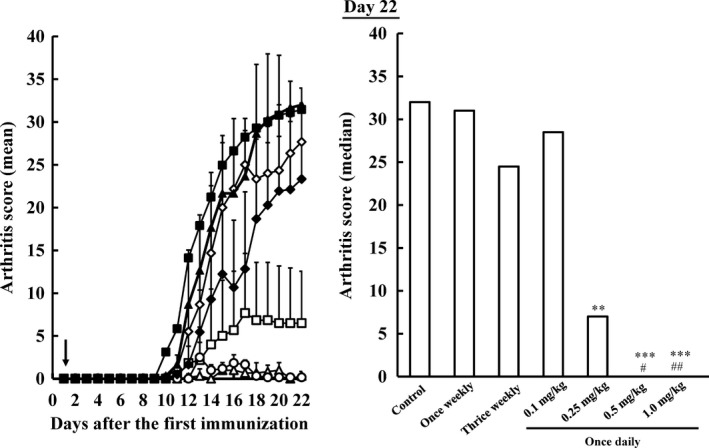
Influence of the dosage of methotrexate on arthritis scores in collagen‐induced arthritis rats when methotrexate was administered once a day. Methotrexate was orally administered at a dose of 0.1 (open diamonds), 0.25 (open squares), 0.5 (open triangles) and 1.0 (open circles) mg/kg once daily. Methotrexate was orally administered at a dose of 7.0 mg/kg once weekly (closed triangles) and 2.33 mg/kg thrice weekly (closed diamonds). Sodium bicarbonate was administered to the control group (closed squares). Each value represents the mean ± SD (*n* = 6–18). An arrow showed start date of methotrexate administration. **P < 0.01 and ***P < 0.001 vs the control group, ^#^ *P* < 0.05 and ^##^ *P* < 0.01 vs the once‐weekly group. Although the total dose per week in the 0.25 mg/kg once‐daily group was one‐quarter that in the thrice‐weekly group, arthritis scores in 0.25 mg/kg once‐daily group were markedly lower than in the thrice‐weekly group.

Collagen‐induced arthritis rats were orally administered MTX 2.33 mg/kg thrice weekly, 7.0 mg/kg once weekly and 0.1, 0.25, 0.5 and 1.0 mg/kg once daily for 3 weeks, and arthritis scores were measured (Figure 3). On day 22, arthritis scores (mean ± SD) were 31.4 ± 0.7 in the control group, 32.0 ± 2.0 in the once‐weekly group, 23.3 ± 8.9 in the thrice‐weekly group, 27.7 ± 3.7 in the 0.1 mg/kg once‐daily group, 6.5 ± 6.1 in the 0.25 mg/kg once‐daily group, 0.3 ± 0.5 in the 0.5 mg/kg once‐daily group and 0.2 ± 0.4 in the 1.0 mg/kg once‐daily group. The aggravation of joint inflammation was not inhibited significantly more in the thrice‐weekly and once‐weekly groups than in the control group. Arthritis scores were significantly lower in the 0.25, 0.5 and 1.0 mg/kg once‐daily groups than in the control groups. The inhibitory effects of MTX on arthritis occurred in a dose‐dependent manner.

### Influence of the dosing schedule of methotrexate on serum type II collagen antibody levels in collagen‐induced arthritis rats

**Figure 4:**
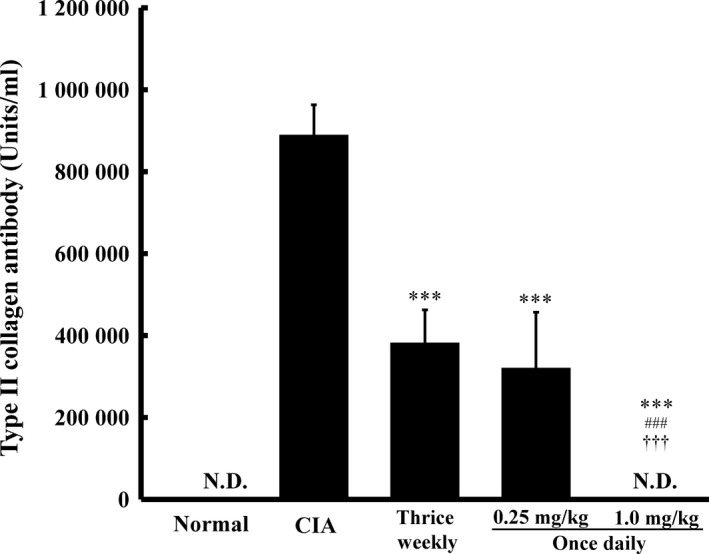
Influence of the dosing schedule of methotrexate on serum type II collagen antibody levels in collagen‐induced arthritis rats. Methotrexate was orally administered thrice weekly (2.33 mg/kg per time) and once daily (0.25 or 1.0 mg/kg) for 3 weeks. Sodium bicarbonate was administered to the normal group and collagen‐induced arthritis groups. Each value represents the mean ± SD (*n* = 6). ***P < 0.001 vs the collagen‐induced arthritis group, ^###^ *P* < 0.001 vs the thrice‐weekly group, ^†††^ *P* < 0.001 vs the 0.25 mg/kg once‐daily group. Serum type II collagen antibody levels were similar between the 0.25 mg/kg once‐daily (1.75 mg/kg per week) and 2.33 mg/kg thrice‐weekly (7 mg/kg per week) groups. Type II collagen antibody levels were below the detection limit in the 1.0 mg/kg once‐daily group, in which the inhibition of arthritis was the greatest.

Collagen‐induced arthritis rats were orally administered MTX thrice weekly (2.33 mg/kg) or once daily (0.25 or 1.0 mg/kg) for 3 weeks, and serum anticollagen antibody levels were measured on day 22 (Figure 4). Serum anticollagen antibody levels in nonimmunized and 1.0 mg/kg once‐daily groups were below the detection limit. Serum anticollagen antibody levels were significantly lower in the thrice‐weekly and once‐daily (0.25 and 1.0 mg/kg) groups than in the CIA group (*P* < 0.001, respectively). No significant differences were observed in serum anticollagen antibody levels between the thrice‐weekly and 0.25 mg/kg once‐daily groups. Serum anticollagen antibody levels were significantly lower in the 1.0 mg/kg once‐daily group than in the thrice‐weekly and 0.25 mg/kg once‐daily groups (*P* < 0.001, respectively).

### Influence of the dosing schedule of methotrexate on adverse effects

**Figure 5:**
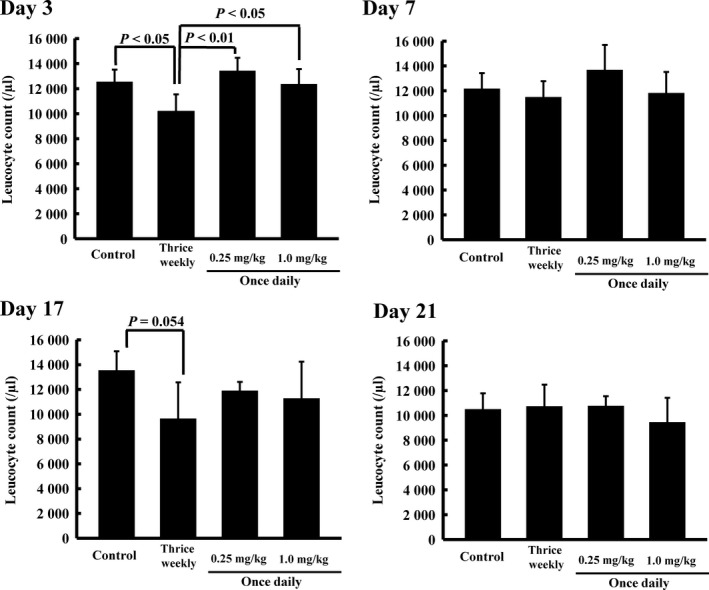
Influence of the dosing schedule of methotrexate on leucocyte counts on days 3, 7, 17 and 21. Methotrexate was orally administered thrice weekly (2.33 mg/kg per time) and once daily (0.25 mg/kg or 1.0 mg/kg) for 3 weeks. Sodium bicarbonate was administered to the normal group and collagen‐induced arthritis groups. Each value represents the mean ± SD (*n* = 6). On day 3, leucocyte counts in the thrice‐weekly group were significantly lower than those in the other groups (*P* < 0.05 and <0.01, respectively).

Normal rats were orally administered MTX thrice weekly (2.33 mg/kg) or once daily (0.25 or 1.0 mg/kg) for 3 weeks. On day 3, leucocyte counts were significantly lower in the thrice‐weekly group than in the control and once‐daily (0.25 and 1.0 mg/kg) groups (*P* < 0.05 and *P* < 0.01, respectively; Figure 5). On day 17, the thrice‐weekly group showed myelosuppression (*P* = 0.054). On days 7 and 21, no significant differences were observed in leucocyte counts among any of the dosing groups.

Alanine aminotransferase and BUN serum concentrations were measured, and no significant differences were observed between any of the groups (Table 2).

**Table 2:** Influence of the dosing schedule of methotrexate on alanine aminotransferase (ALT) and blood urea nitrogen (BUN) concentrations on days 7, 14 and 21

	Group	Day 7	Day 14	Day 21
ALT (Karmen units)	Control	32.66 ± 5.62	39.07 ± 2.76	38.04 ± 5.21
	Thrice weekly	35.99 ± 3.19	42.05 ± 3.21	42.77 ± 1.47
	Once daily			
	0.25 mg/kg	38.03 ± 6.14	33.29 ± 4.19	43.26 ± 2.73
	1.0 mg/kg	36.73 ± 2.77	29.04 ± 7.96	41.68 ± 3.24
BUN (mg/dl)	Control	20.54 ± 2.09	20.83 ± 1.21	18.71 ± 1.92
	Thrice weekly	20.98 ± 1.87	20.37 ± 1.25	22.21 ± 2.70
	Once daily			
	0.25 mg/kg	20.45 ± 2.20	22.00 ± 1.92	22.69 ± 0.40
	1.0 mg/kg	20.85 ± 0.91	19.22 ± 2.17	21.72 ± 2.28

Each value represents the mean ± SD (*n* = 6).

## Discussion

The inhibition of arthritis was significantly greater in the MTX‐treated group than in the control group when CIA rats received 0.1 mg/kg MTX orally once a day.^13^ However, this dosage did not lead to the development of adverse effects in the MTX group. The daily administration of MTX is generally not considered to protect patients with RA from the adverse effects associated with this drug. Thus, suitable dosages need to be selected in order to estimate its efficacy and toxicity. In the present study, we selected a total dose of 7.0 mg/kg per week because rats treated MTX once a day did not show toxic death and exhibited antirheumatic effects.

Collagen‐induced arthritis represents a true autoimmune reaction against major joint components with the association of class II major histocompatibility complex genes and pannus formation. The CIA model is similar to RA in terms of its pathology, immunology and genetics.^14^, ^15^ After the third sensitization of CII, all rats developed arthritis after a few days, and most rats reached maximum arthritis scores for approximately 1 week. One of the characteristics of the CIA model is that arthritis score is the greatest at about 10 days after the onset of arthritis. The aggravation of arthritis was not inhibited in any of the dosing schedule groups even though MTX (14.0 mg/kg per week) was administered from day 11 after the first sensitization (Figure S1). The MTX‐treated groups did not exhibit any antirheumatic effects over the control group even though the dosage administered in the present study was 10‐fold higher than that in a previous study.^13^ This may have been because the dose selected was insufficient to inhibit fulminant arthritis because the administration of MTX was initiated in CIA rats from a few days after the onset of arthritis. In a previous study, MTX was administered from the next day after the first sensitization.^13^ Therefore, we estimated the influence of the dosing schedule on antirheumatic effects during the administration of MTX from the next day after the first or second sensitization.

In the two experiments, the daily administration of a low dose of MTX exerted the greatest inhibitory effects on the aggravation of arthritis among all of the groups examined regardless of the different initiation times of MTX after the first sensitization. Arthritis did not develop in the once‐daily group only, which was administered 1 mg/kg of MTX daily for 21 days from the next day after the first sensitization. Arthritis scores were measured from day 12, and markedly increased for a few days in the present study. The total doses of MTX in the once‐ and thrice‐weekly groups on the last sensitization day (day 10) were 14 and 7 mg/kg, whereas those in the once‐daily group were 10 and 4 mg/kg when MTX was administered after the first and second sensitization. On day 12, the time at which arthritis began to be observed, the total dose in the once‐daily group was lower than those in the other MTX‐treated groups.

The thrice‐weekly group showed a transient suppressing effect on arthritis when MTX was administered after the first sensitization. Arthritis scores were considered to decrease because the third treatment was started on day 15. However, arthritis in the thrice‐weekly group rapidly became aggravated during the washout period after the administration of MTX. It might be caused by a failure to suppress inflammation due to the washout. On the other hand, arthritis scores (mean ± SD) were 31.3 ± 0.8 in the control group, 30.5 ± 2.0 in the once‐weekly group, 27.0 ± 5.9 in the thrice‐weekly group and 0.3 ± 0.5 in the once‐daily group when measured 7 days after the last administration of MTX (day 29). The aggravation of arthritis continued to be inhibited in the once‐daily group (*P* < 0.001; Figure S2). Therefore, greater therapeutic effects were achieved with the daily administration of a low dose of MTX than with its weekly administration at a high dose when the total dosage per week was the same.

Once joint destruction begins in RA, it is irreversible, and the difficulties and challenges encountered in everyday life by patients with RA as a result are immeasurable. Thus, the success of RA therapy depends on its initiation during the early stages of the disease.^16^ In the present study, joint inflammation was not inhibited in the thrice‐weekly and once‐weekly groups despite the initiation of the MTX treatment performed in early stage of RA onset. However, the increase in arthritis was markedly improved in the once‐daily group. These results indicate that the initiation of the MTX treatment as early as possible after the onset of RA and its administration every day is beneficial for achieving greater antirheumatic effects.

The daily administration of the low dose of MTX achieved greater antirheumatic effects than the current standard dosing method. However, a method to restrict the dosage of MTX as much as possible and obtain high curative effects is more advantageous for patients with RA to ensure safety because MTX has been used as an anticancer agent. Therefore, we examined the influence of the dosage of MTX on antirheumatic effects when it was administered once daily for 21 days from the next day after the first sensitization. The control of joint inflammation was significantly better in the 0.25, 0.5 and 1.0 mg/kg once‐daily groups than in the control, thrice‐weekly, once‐weekly and 0.1 mg/kg once‐daily groups. Furthermore, the once‐daily group showed dose‐dependent arthritis‐suppressing effects. Even though the dose per week in the 0.25 mg/kg once‐daily group was one‐quarter that in the thrice‐weekly and once‐weekly groups, arthritis scores in the once‐daily group 1/5 to 2/7 lower than those in these groups. These results demonstrate that the selection of a suitable schedule reduces the total dose per week and achieves greater antirheumatic effects.

The CIA model rat is immunized with cow type II collagen, and arthritis is induced using a cross‐reaction. Previous studies have reported that antitype II collagen antibody levels decrease when a joint inflammation model is administered MTX.^17^, ^18^ In this study, antitype II collagen antibody levels were markedly higher in the sensitization group than in the nonsensitization group. Increases in antitype II collagen antibody levels were significantly inhibited in the thrice‐weekly and once‐daily groups. Furthermore, the antitype II collagen antibody was not detected in the 1.0 mg/kg once‐daily group. These results revealed dose‐dependent changes in arthritis scores and antitype II collagen antibody levels in the once‐daily groups. However, although the 0.25 mg/kg once‐daily group had significantly lower arthritis scores than those in the thrice‐weekly group, antibody levels were not significantly different. Thus, the suppression of joint inflammation may not necessarily be related to the inhibitory effects of the antitype II collagen antibody.

The contribution of anti‐inflammatory actions due to the participation of adenosine may also be of importance. Adenosine is an endogenous anti‐inflammatory factor thereby contributing to the progression of arthritis. A previous study reported that MTX acts as a 5‐aminoimidazole‐4‐carboxamide ribonucleotide repressor and increases adenosine levels.^19^ Adenosine suppresses neutrophil migration to areas of inflammation, promotes the differentiation of macrophages and also inhibits the production of interleukin‐1 or leukotrienes B4. The mechanism of action of MTX may involve the suppression of joint inflammation by not only immunosuppressive actions but also increasing adenosine levels. The administration of a low dose of MTX (0.25 mg/kg per week) suppressed the aggravation of arthritis significantly more than standard dosing schedules (7 mg/kg per week) despite both groups having similar collagen antibody levels, and this may be due to their effects on adenosine.

In the present study, the greatest arthritis‐suppressing effect was achieved when MTX was administered every day. However, this treatment may lead to the development of severe adverse effects. Therefore, we evaluated toxicity. Liver and renal functions were normal in all treated groups. Myelosuppression was observed on days 3 and 17 in the thrice‐weekly group. After the washout period of MTX, leucocyte counts recovered to normal levels. On the other hand, myelosuppression did not appear when MTX was administered every day. The daily administration of MTX in RA therapy is generally considered to enhance the risk of adverse effects. However, the daily administration of a low dose of MTX maintained safety, whereas the thrice‐weekly administration of a high dose temporarily resulted in leukopenia, even though the total amount administered in 1 week was the same. A sudden decrease in leucocyte counts reduces immune function and may increase the risk of infectious diseases. Therefore, the risk of toxicity caused by MTX is considered to be higher with the current standard dosing method than with its daily administration.

Despite the same dosage per week, there is a possibility that bioavailability is relatively higher in the daily administration group than in the other groups as a factor that causes a significant difference in the arthritis suppression effect. However, transient myelosuppression was shown in the thrice‐weekly group, but no toxicity was observed for 21 days in the daily administration group with high therapeutic effect. Considering that the relative increase of bioavailability affected the difference in treatment effect, it cannot explain sufficiently the consistency with the difference toxicity. Thus, we thought that the dosing schedule is highly involved in the success or failure of treatment if the dosage of MTX is the same amount.

A number of studies have attempted to improve the safety of treatments with MTX. Chronopharmacology is one of the methods used to achieve this, and the selection of an optimal dosing schedule of MTX was previously reported to be essential for yielding an excellent therapeutic index in basic and clinical studies.^8^, ^12^, ^20^ In this clinical study, patients with RA were administered MTX once daily at bedtime. Therefore, chronotherapy in addition to the daily administration of MTX used in the present study will lead to more effective and safer treatments for RA.

## Conclusions

Arthritis‐suppressing effects were the greatest in the once‐daily group in this present study. Moreover, the maintenance of these effects was better in the once‐daily group than in the other groups even though the dosage administered was lower. We also demonstrated that the high curative effects in the once‐daily group were achieved safely. We revealed that the daily administration of a low dose of MTX exerted strong inhibitory effects on arthritis better than the current standard dosing methods, and, thus, has potential as an RA therapy. We are now performing a clinical trial to confirm our results.

## Declarations

### Conflict of interest

The Authors declare that they have no conflict of interests to disclose.

### Funding

This study was supported by the Japan Research Foundation for Clinical Pharmacology.

## Supporting information


**Figure S1.** MTX was orally administered at a dose of 14.0 mg/kg once weekly (*n* = 6), 4.66 mg/kg thrice weekly (*n* = 6), and 2.0 mg/kg once daily (*n* = 6) for 7 days from the next day after the third immunization (day 11).

**Figure S2.** MTX was orally administered at a dose of 7.0 mg/kg once weekly (*n* = 6), 2.33 mg/kg thrice weekly (*n* = 6), and 1.0 mg/kg once daily (*n* = 6) for 3 weeks from the next day after the first immunization.
