# Supplementary figures and images for: Daily oral administration of low‐dose methotrexate has greater antirheumatic effects in collagen‐induced arthritis rats
Source: J Pharm Pharmacol. 2017 May 31;69(9):1145–54. doi: 10.1111/jphp.12752 (PMC5575561; doi:10.1111/jphp.12752)

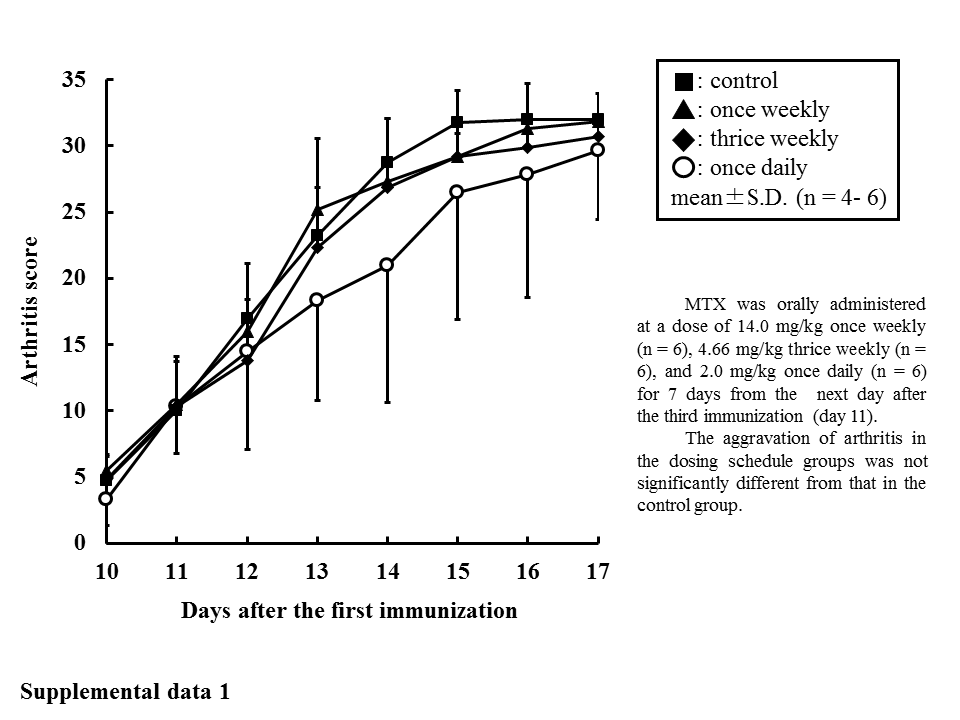

Supplement: Supplementary file 1 — Figure S1. MTX was orally administered at a dose of 14.0 mg/kg once weekly (n = 6), 4.66 mg/kg thrice weekly (n = 6), and 2.0 mg/kg once daily (n = 6) for 7 days from the next day after the third immunization (day 11). [file JPHP-69-1145-s001.TIF]

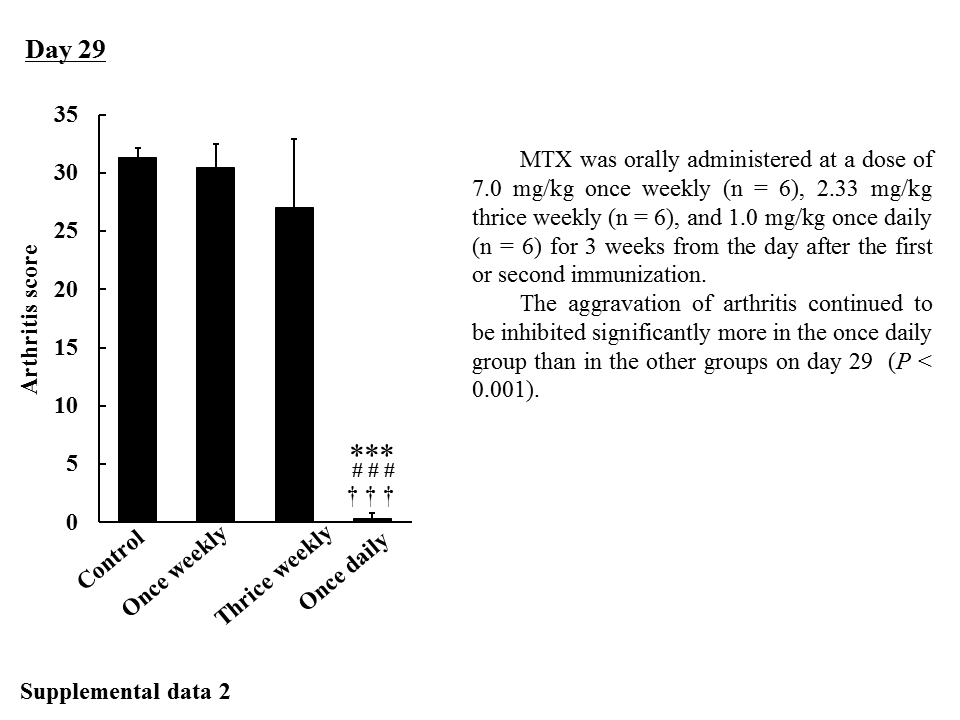

Supplement: Supplementary file 2 — Figure S2. MTX was orally administered at a dose of 7.0 mg/kg once weekly (n = 6), 2.33 mg/kg thrice weekly (n = 6), and 1.0 mg/kg once daily (n = 6) for 3 weeks from the next day after the first immunization. [file JPHP-69-1145-s002.TIF]
